# *Allomyrina dichotoma* (Arthropoda: Insecta) Larvae Confer Resistance to Obesity in Mice Fed a High-Fat Diet

**DOI:** 10.3390/nu7031978

**Published:** 2015-03-17

**Authors:** Young-Il Yoon, Mi Yeon Chung, Jae-Sam Hwang, Myung Sae Han, Tae-Won Goo, Eun-Young Yun

**Affiliations:** 1 Department of Agricultural Biology, National Academy of Agricultural Science, RDA, Wanju-gun 565-851, Korea; E-Mails: ruru012012@gmail.com (Y.-I.Y.); shindelela@hanmail.net (M.Y.C.); hwangjs@korea.kr (J.-S.H.); 2 Department of Bio-fibers and Materials Science, Kyungpook National University, Daegu 702-701, Korea; E-Mail: mshan@knu.ac.kr; 3 Department of Biochemistry, School of Medicine, Dongguk University, Gyeongju 780-714, Korea

**Keywords:** high-fat diet (HFD), *Allomyrina dichotoma*, adipogenesis, lipogenesis, obesity

## Abstract

To clarify the anti-obesity effect of *Allomyrina dichotoma* larvae (ADL), we previously reported that ADL block adipocyte differentiation on 3T3-L1 cell lines through downregulation of transcription factors, such as peroxisome proliferator-activated receptor-γ (PPARG) and CCAAT/enhancer binding protein-α (CEBPA). In this study, we tested whether ADL prevent obesity in mice fed a high-fat diet (HFD) and further investigated the mechanism underlying the effects of ADL. All mice were maintained on a normal-fat diet (NFD) for 1 week and then assigned to one of five treatment groups: (1) NFD; (2) HFD; (3) HFD and 100 mg·kg^−1^·day^−1^ ADL; (4) HFD and 3000 mg·kg^−1^·day^−1^ADL; or (5) HFD and 3000 mg·kg^−1^·day^−1^ yerba mate (*Ilex paraguariensis*, positive control). ADL and yerba mate were administered orally daily. Mice were fed experimental diets and body weight was monitored weekly for 6 weeks. Our results indicated that ADL reduced body weight gain, organ weight and adipose tissue volume in a dose-dependent manner. Body weight gain was approximately 22.4% lower compared to mice fed only HFD, but the difference did not reach the level of statistical significance. Real-time polymerase chain reaction (PCR) analysis revealed that gene expression levels of PPARG, CEBPA and lipoprotein lipase (LPL) in the epididymal fat tissue of HFD-fed mice receiving 3000 mg·kg^−1^·day^−1^ ADL were reduced by 12.4-, 25.7-, and 12.3-fold, respectively, compared to mice fed HFD only. Moreover, mice administered ADL had lower serum levels of triglycerides and leptin than HFD-fed mice that did not receive ADL. Taken together our results suggest that ADL and its constituent bioactive compounds hold potential for the treatment and prevention of obesity.

## 1. Introduction

Obesity is a chronic metabolic disease caused by an imbalance of energy intake and expenditure [1]. The prevalence of obesity is a public health issue, because it is a major risk factor for hyperlipidemia, diabetes mellitus, hypertension, atherosclerosis, cardiovascular disease and certain types of cancer [2]. In addition, the accumulation of excess fat in obesity increases the number and size of adipocytes derived from fibroblastic preadipocytes in adipose tissue [3]. Pharmacological approaches for the treatment and control of obesity have included drugs that suppress appetite, inhibit nutrient absorption and otherwise promote weight loss [4,5]. To effectively treat for obesity-related diseases, it is also important to prevent differentiation that leads to an increase in the number of mature adipocytes [6]. However, owing to the adverse side effects associated with many anti-obesity drugs, more recent trials have focused on natural compounds reported to have minimal side effects [7]. Although crude extracts and compounds isolated from plants have been studied to treat obesity [8,9], and interest in insect-based bioactive compounds has recently grown [10], few insect-derived compounds have been studied in relation to obesity until now.

Recently, the Food and Agriculture Organization of the United Nations (FAO) reported the utility of edible insects as human dietary supplements. These are already used as traditional foods in many parts of world and are a rich source of fats, minerals, vitamins and other nutrients [11,12,13,14]. The Korean horn beetle (*Allomyrina dichotoma*) is recognized in traditional medicine to have anti-hepatofibrotic, anti-neoplastic and antibiotic effects [15,16,17]. In a recent study, we reported that *A. dichotoma* larvae (ADL) inhibited *in vitro* differentiation of 3T3-L1 preadipocytes via downregulation of transcription factors, such as peroxisome proliferator-activated receptor-γ (PPARG) and CCAAT/enhancer binding protein-α (CEBPA) [18]. The murine 3T3-L1 cell line is one of most reliable models for preadipocyte-to-adipocyte differentiation [19].

Thus, in the present study, we extended our *in vitro* finding to a live animal model to investigate the effects of ADL on HFD-induced obesity. Obesity studies commonly employ high-fat diets to induce visceral obesity in rodent animal models, in which the pathogenesis of obesity is similar to that in humans [20,21,22]. Therefore, the high-fat diet animal model of obesity was selected for this study. We analyzed body weight changes, serum biomarkers of obesity and histology in mice fed normal- or high-fat diets, with or without ADL or *Ilex paraguariensis* (yerba mate)—which has known anti-obesity effects in animals via decrease of body weight gain, adipokines mRNA levels, serum triglyceride (TG) and low density lipoprotein (LDL) cholesterol level [23]—was used as a positive control. Additionally, to elucidate the molecular mechanism underlying the effects of ADL, we investigated the differential expression of genes related to lipid metabolism. Our results demonstrate the potential of ADL as a novel treatment option for obesity.

## 2. Materials and Methods

### 2.1. Preparation of A. dichotoma Larvae (ADL)

Third instar *A. dichotoma* larvae were purchased from Canaan farm (Gapyeong-gun, Gyungsangnam-do, Korea). Lyophilized yerba mater (MT) leaf and ADL were ground into powder and suspended in deionized water. The sample concentrations were adjusted to 100 mg·kg^−1^·day^−1^ (ADL) and 3000 mg·kg^−1^·day^−1^ (MT and ADL).

### 2.2. Animals and Diets

This experimental design was approved by the Institutional Animal Care and Use Committee (IACUC) of the National Academy of Agricultural Science (NAAS-1403). Five-week-old male BALB/c mice were supplied by Central Lab Animal, Inc. (Seoul, Korea) and maintained at ambient temperature (22 ± 1 °C) with 12:12 h light-dark cycles and free access to water and feed. At six weeks of age, the mice were randomly assigned to one of five treatment conditions: (1) NFD; (2) HFD; (3) HFD with 100 mg·kg^−1^·day^−1^ ADL, (4) HFD with 3000 mg·kg^−1^·day^−1^ ADL, and (5) HFD with 3000 mg·kg^−1^·day^−1^ MT. Each treatment condition consisted of seven mice (*n* = 7). The normal-fat diet (Research Diet Inc., New Brunswick, NJ, USA) provided approximately 3.85 kcal/g with a macronutrient profile of 20% protein, 70% carbohydrate and 10% fat. The high-fat diet (Research Diet Inc., NJ, USA) provided 4.73 kcal/g with a macronutrient profile of 20% protein, 35% carbohydrate, and 45% fat. Mice were fed with NFD or HFD for 6 weeks. ADL (100 mg/kg or 3000 mg/kg) and MT (3000 mg/kg) were administered daily by oral gavage using disposable mouse feeding needle (Scientific Hub Services Pte Ltd., Singapore, Singapore). Body weight was measured weekly and weight gain was calculated as the difference between initial and final body weights, divided by initial body weight. At the end of the experimental period, the randomly selected mice (*n* = 3) were fasted for 15 h prior to sacrifice. Immediately following cervical spine dislocation, the liver, epididymal adipose tissue and abdominal-to-peripheral adipose tissue were dissected, weighed and frozen at −80 °C.

### 2.3. Histological Analysis

After draining the blood, the liver and epididymal adipose tissue were fixed in 10% neutral formalin solution for 48 h. Tissues were subsequently dehydrated in a graded ethanol series (75%–100%) and embedded in paraffin wax. The embedded tissue was sectioned (8 μm), stained with hematoxylin and eosin (H & E), examined by light microscopy (Leica CTR6000, Hesse, Germany), and photographed. Epididymal adipose cell volume was estimated using the IMT i-Solution Lite (version 8.0, IMT i-Solution Inc., Northampton, NY, USA).

### 2.4. RNA Preparation and Quantitative Real-Time PCR Analysis

Total RNA was isolated from epididymal adipose tissue using Trizol reagent (Invitrogen, Carlsbad, CA, USA) and RNA concentration and purity were measured using a UV/Vis spectrophotometer (Beckman Coulter Co., Miami, FL, USA). Complementary DNA (cDNA) was synthesized from 1 μg of total RNA using the High Capacity cDNA Reverse Transcription Kit (Applied Biosystems, Foster City, CA, USA). Real-time polymerase chain reaction (PCR) amplification was performed with Power SYBR Green Master Mix using a 7500 Real-Time PCR System (both from Applied Biosystems), according to the manufacturer’s instructions. The PCR program was as follows: an initial temperature of 95 °C for 5 min followed by 35 thermal cycles of 94 °C for 30 s, 60 °C for 30 s and 72 °C for 30 s. For detection of target gene transcripts, we designed specific forward and reverse oligonucleotide primers using Beacon Designer software (PREMIER Biosoft, Palo Alto, CA, USA). The primers sequences are listed in Table 1. Target mRNA levels were normalized using glyceraldehyde 3-phosphate dehydrogenase (*GAPDH*) as an internal control to quantify the relative expression of target mRNA according to the cycling threshold (ΔCt) method. All samples were analyzed in triplicate.

**Table 1:** Primer sequences for amplification of genes involved in adipose metabolism and for GAPDH internal standard.

Gene Symbol	Gene Name	Primer Sequence
PPARG	Peroxisome proliferator-activated receptor-γ	Forward: 5′-TGGGAACCTGGAAGCTTGTCTC-3′
		Reverse: 5′-TGTGGTAAAGGGCTTGATGT-3′
C/EBPA	CCAAT/enhancer-binding protein-α	Forward: 5′-TGCCTATGAGCACTTCACAA-3′
		Reverse:5′-AACTCCAGCACCTTCTGTTG-3′
LPL	Lipoprotein lipase	Forward: 5′-TCCAAGGAAGCCTTTGAGAA-3′
		Reverse: 5′-CCATCCTCAGTCCCAGAAAA-3′
GAPDH	Glyceraldehyde-3-phosphate dehydrogenase	Forward: 5′-CTGGAGAAACCTGCCAAGTA-3′
		Reverse: 5′-AGTGGGAGTTGCTGTTGAAG-3′

### 2.5. Biochemical Analysis

Blood samples were obtained from mice at the end of the experimental period after having been fasting for 15 h. Samples were centrifuged at 3000 rpm for 15 min at 4 °C and serum samples were stored at −70 °C. Serum triglyceride (TG), total cholesterol (TC), high density cholesterol (HDL-C) and low density cholesterol (LDL-C) levels were assayed using enzymatic colorimetric assay kits (Roche, Mannheim, Germany) and modular analytics (Roche, Mannheim, Germany). Serum concentrations of leptin were measured with mouse enzyme-linked immunosorbent assay (ELISA) kits (R & D systems, Minneapolis, MN, USA) according to the manufacturer’s instructions. Absorbance was measured using the microplate spectrophotometer, as mentioned above.

### 2.6. Statistical Analysis

Data were presented as averages with standard deviations (mean ± SD) from at least three independent experimental replicates. Statistical analyses employed the R statistical software (version 3.03, Lucent Technologies Inc., New Providence, NJ, USA). Differences among groups were evaluated using Duncan’s post-hoc test of analysis of variance (ANOVA). Differences were considered statistically significant at *p* < 0.05.

## 3. Results and Discussion

### 3.1. Effect of ADL on Body Weight and Adipose Tissue Weight

To investigate the anti-obesity effects of ADL *in vivo*, mice were fed either a normal-fat diet or a high-fat diet for six weeks. Mice in the NFD and HFD treatment groups also received no supplement or daily doses of 100 mg/kg ADL, 3000 mg/kg ADL, or 3000 mg/kg MT. Body weight was monitored weekly. Results revealed that the final mean body weight gain for HFD mice (receiving no supplement) was about 90% higher than that of NFD mice. Intriguingly, oral administration of ADL reduced body weight gain induced by HFD for all doses. For mice in the HFD + ADL (3000 mg·kg^−1^·day^−1^) treatment group, weight gain was approximately 22.4% lower compared to mice fed only HFD (Table 2).

**Table 2:** Effects of *Allomirina dichotoma* larva (ADL) on body weight gain.

Group	Body Weight (g)		Weight Gain (g)
	Initial	Final	
NFD (*n* = 7)	32.95 ± 1.33	46.07 ± 3.28	0.40 ± 0.13
HFD (*n* = 7)	32.01 ± 1.37	56.53 ± 7.64	0.76 ± 0.29
HFD + ADL 100 (*n* = 7)	32.26 ± 2.11	54.47 ± 7.30	0.69 ± 0.29
HFD + ADL 3000 (*n* = 7)	31.66 ± 1.20	50.31 ± 5.85	0.59 ± 0.22
HFD + MT 3000 (*n* = 7)	32.10 ± 1.33	45.72 ± 4.74 *	0.42 ± 0.18 *

NFD: Normal-fat diet, HFD: high-fat diet group, HFD + ADL 100: high-fat diet and 100 mg·kg^−1^·day^−1^ ADL, HFD + ADL 3000: high-fat diet and 3000 mg·kg^−1^·day^−1^ ADL, HFD + MT 3000: high-fat diet and 3000 mg·kg^−1^·day^−1^ yerba mate. Weight gain (g) = (Final body weight − Initial body weight)/Initial body weight. Values represent mean ± SD (*n* = 7). * *p* < 0.05, compared to the high-fat diet group.

To investigate whether ADL decreases visceral and peripheral adiposity (excessive body fat), mice were sacrificed and the livers and epididymal and abdominal-to-peripheral adipose tissues were removed and weighed. Epididymal adipose tissue is an important marker of visceral adiposity in mice [24]. We found that epididymal and abdominal-to-peripheral adipose tissue weights were 42.9% and 16.3% lower, respectively, in the group fed HFD + ADL (3000 mg·kg^−1^·day^−1^) than in the HFD group (Table 3). However, liver adipose tissue weight showed little difference (data not shown). In the positive control mice fed HFD + MT, abdominal-to-peripheral adipose tissue weight was approximately 42.9% lower than that of the HFD group, while epididymal adipose tissue weight was 3.9% higher. This result is consistent with previous reports that show that crude MT suspension does not significantly decrease epididymal adipose tissue weight in rats [25,26]. Although ADL was similar to MT in its effects on abdominal-to-peripheral adipose tissue, it also succeeded in reducing epididymal adipose tissue, which is resistant to MT.

In obese individuals, adipocytes expand to store more energy. Recent studies reveal that adipocytes are not simply storage depots for energy, but also secrete endocrine factors such as adipokine, cytokine and growth factor [27,28]. Once adipocytes reach their maximum capacity for energy storage, they can recruit macrophages, promote inflammation and lead to obesity-induced insulin resistance. This is important for understanding the pathogenesis of obesity-induced chronic inflammation, certain types of tumor and type II diabetes. Therefore, reducing adipocyte size appears critical for the treatment of obesity and associated diseases [29,30].

**Table 3:** Effects of *Allomirina dichotoma* larva (ADL) on relative organ weight.

Group	Abdominal-to-Peripheral Fat	Epididymal Fat
NFD (*n* = 3)	3.55 ± 1.5	0.177 ± 0.01
HFD (*n* = 3)	7.52 ± 1.5	0.178 ± 0.03
HFD + ADL 100 (*n* = 3)	6.28 ± 1.8	0.220 ± 0.04
HFD + ADL 3000 (*n* = 3)	4.29 ± 1.8 *	0.149 ± 0.02 *
HFD + MT 3000 (*n* = 3)	4.29 ± 0.9 *	0.185 ± 0.02

NFD: Normal-fat diet, HFD: high-fat diet group, HFD + ADL 100: high-fat diet and 100 mg·kg^−1^·day^−1^ ADL, HFD + ADL 3000: high-fat diet and 3000 mg·kg^−1^·day^−1^ ADL, HFD + MT 3000: high-fat diet and 3000 mg·kg^−1^·day^−1^ yerba mate. Values represent mean ± SD (*n* = 3). * *p* < 0.05, compared to the high-fat diet group.

**Figure 1:**
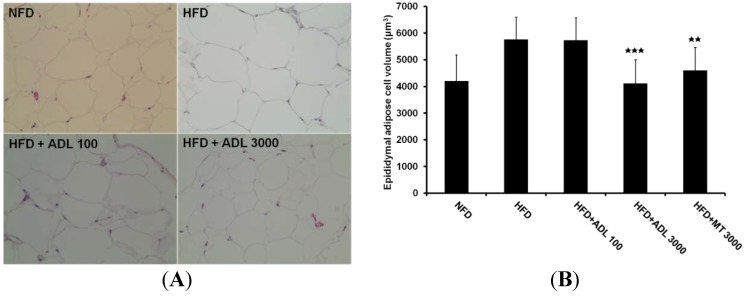

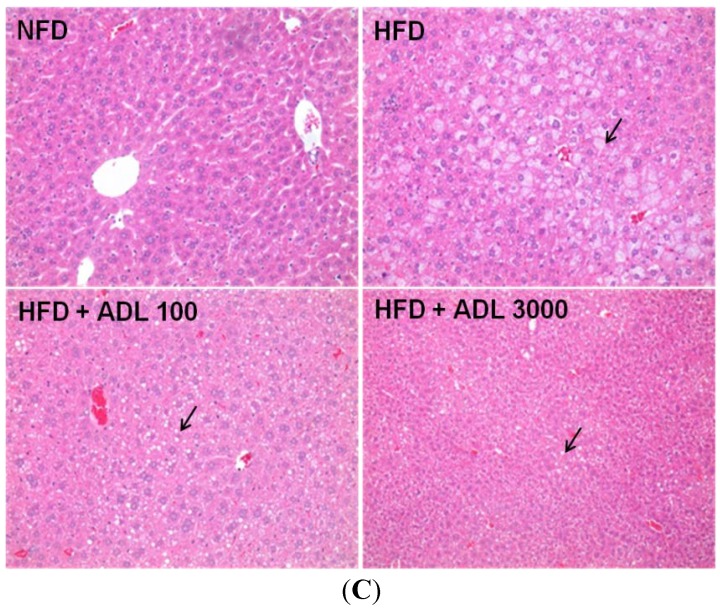
Histology of epididymal adipose (**A**) and liver (**C**) tissues of mice fed experimental diets for six weeks (magnification= ×200). Normal-fat diet (NFD), high-fat diet (HFD), high-fat diet with 100 mg·kg^−1^·day^−1^ *Allomirina dichotoma* larva suspension (HFD + ADL 100), and HFD with 3000 mg·kg^−1^·day^−1^ ADL (HFD + ADL 3000). Epididymal adipose cell volume measured by IMT i-Solution Lite program (**B**). Histopathology of the liver with steatosis. Arrows indicate the macrovesicular lipid droplets in the cytoplasm of hepatocytes (**C**). Results are expressed as mean ± SD (*n = *3). Statistical significance was evaluated by Duncan post-hoc test in ANOVA. ^
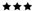
^ *p* < 0.001 and ^
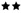
^ *p* < 0.01, compared to the high-fat diet group.

Accordingly, we investigated effects of ADL on adipocyte size. Our results show that adipocytes from epididymal white adipose tissue of mice fed HFD are significantly reduced in size by oral administration of ADL, and that this effect is dose-dependent (Figure 1A,B). Mean adipocyte volumes in HFD, HFD + ADL (3000 mg·kg^−1^·day^−1^) and HFD + MT mice were 5760 μm^3^, 4115 μm^3^ and 4600 μm^3^, respectively. In addition, fat accumulation and cell size in liver tissue were reduced in groups receiving HFD + ADL compared to the HFD group (Figure 1C). Macrovesicular steatosis is the most common form related with alcohol, diabetes, obesity and corticosteroids [31,32]. Macrovesicular steatosis increased in liver of the HFD group was decreased by oral administration of ADL (3000 mg·kg^−1^·day^−1^) (Figure 1C).

In previous studies, we investigated the nutrient profiles of larvae of *Tenebrio molitor* (mealworm) [13] and *Protaetia brevitarsis* (white-spotted flower chafer) [14]. *T*. *molitor* and *P*. *brevitarsis* larvae consisted of 50.3% and 57.9% protein, 33.7% and 16.6% total fat, 3.7% and 8.4% ash, 9.3% and 10.6% carbohydrate, and 2.9% and 6.7% moisture, respectively. It has also been reported that ADL contains 38.1% protein, 32.7% fat, 4.14% ash, 22.7% carbohydrate and 2.3% moisture [33]. On the other hand, the positive control, MT contained 13.2% protein, 7.1% lipid, 6.2% ash, 27.2% total sugar, and 5.8% moisture [34]. Therefore, the ADL administered in this study would have provided greater dietary amounts of both protein and fat than the positive control, MT. ADL may function as a good nutritional source as well as natural anti-obesity treatment.

In summary, our results confirm that ADL reduces body weight gain, adipose tissue weight and volumes of abdominal-to-peripheral and visceral adipose tissues in mice fed high-fat diets.

### 3.2. Inhibitory Effect of ADL on the Expression of Adipogenic and Lipogenic Specific Genes

**Figure 2:**
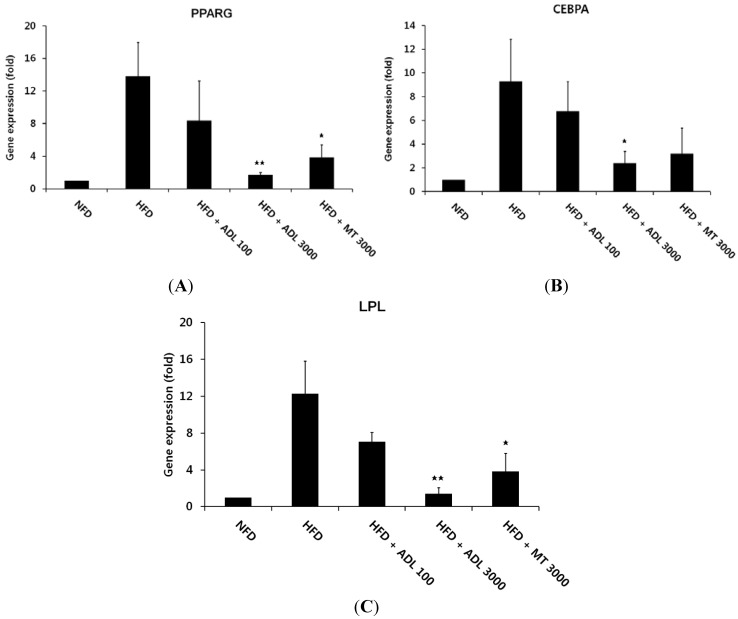
Changes in PPARG (**A**); CEBPA (**B**); and LPL (**C**) gene expression in epididymal adipose tissues of mice fed experimental diets for six weeks was analyzed by real-time PCR. Normal-fat diet (NFD), high-fat diet (HFD), high-fat diet with 100 mg·kg^−1^·day^−1^ *Allomirina dichotoma* larva suspension (HFD + ADL 100), and HFD with 3000 mg·kg^−1^·day^−^^1^ ADL (HFD + ADL 3000). Bars represent mean ± SD (*n *= 3). Statistical significance was evaluated by Duncan post-hoc test in ANOVA. ^
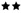
^ *p* < 0.01 and ^
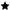
^ *p* < 0.05, compared to the high-fat diet group.

ADL decreased epididymal adipose tissue in HFD-fed mice. To elucidate the mechanism of ADL’s effects, we investigated the mRNA expression levels of adipogenesis- and lipogenesis- related genes in this tissue. The transcription factors PPARG and CEBPA play major roles in the regulation of adipogenesis [35]. Activation of these transcription factors results in terminal differentiation of adipocytes through induction of adipocyte fatty-acid binding protein (aP2), fatty acid synthase (FAS), stearoyl-coenzyme desaturase-1 (SCD1) and lipoprotein lipase (LPL) [36,37]. Compared with the NFD group, the HFD-induced obese mice exhibited higher mRNA levels for PPARG and CEBPA in epididymal adipose tissue. However, administration of ADL (3000 mg·kg^−1^·day^−1^) was associated with 12.4- and 25.7-fold reductions in mRNA expression of PPARG and CEBPA, respectively, compared with the unsupplemented HFD group (Figure 2A,B). We further investigated whether ADL influenced the expression of lipogenic targets such as LPL in epididymal adipose tissue. We found LPL mRNA expression in HFD + ADL (3000 mg·kg^−1^·day^−1^) mice to be 11.2-fold lower than in the unsupplemented HFD group (Figure 2C).

In a previous study, we reported that ADL suspension inhibited differentiation of 3T3-L1 preadipocytes via down-regulation of the transcription factors PPARG, CEBPA and LPL [18]. In accord with this report, here we found that PPARG, CEBPA and LPL expression in epididymal adipose tissue was suppressed in the HFD + ADL group relative to the HFD group. We therefore posit that ADL suppresses the effects of HFD on adipocyte size and epididymal adipose tissue weight via downregulation of these gene products.

### 3.3. Effects of ADL on TG and Leptin Levels in Serum of HFD-Induced Obese Mice

**Figure 3:**
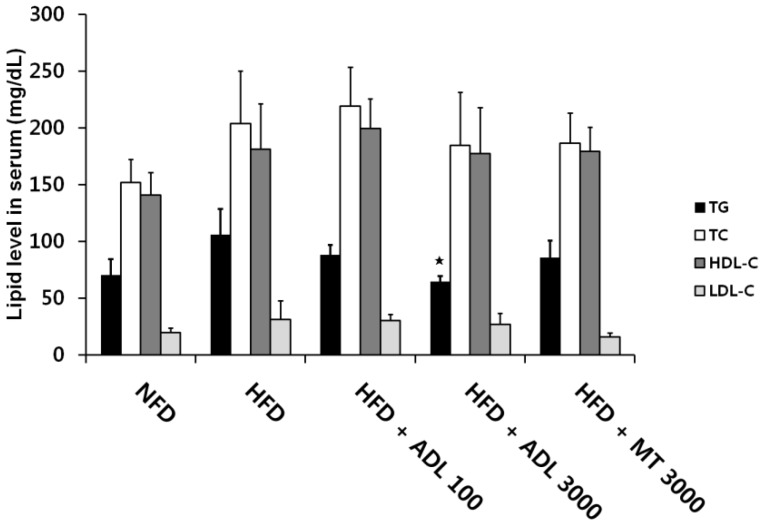
Effects of *Allomirina dichotoma* larva (ADL) suspension on serum triglyceride (TG), total cholesterol (TC), high density lipoprotein cholesterol (HDL-C), and low density lipoprotein cholesterol (LDL-C). Mice were fed normal-fat diet (NFD), high-fat diet (HFD), HFD supplemented with 100 mg·kg^−1^·day^−1^ ADL (HFD + ADL 100), HFD supplemented with 3000 mg·kg^−1^·day^−1^ ADL (HFD + ADL 3000), and HFD supplemented with 3000 mg·kg^−1^·day^−1^ yerba mate (HFD + MT 3000) for 6 weeks. Serum samples were obtained from 15 h-fasted mice. Bars represent mean ± SD (*n *= 3). Statistical significance was evaluated by Duncan post-hoc test in ANOVA. ^
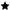
^ *p* < 0.05, compared to the high-fat diet group.

ADL oral administration significantly inhibited the elevated TG levels associated with HFD. However, levels of total cholesterol, HDL-cholesterol and LDL-cholesterol did not differ between the HFD and HFD + ADL mice (Figure 3). In contrast, serum levels of leptin, an adipocytokine, were significantly reduced in HFD + ADL mice relative to HFD mice. It is known that HFD induces TG accumulation in adipocytes via adipogenesis [38]. That is, TG synthesis occurs in mature adipocytes as a marker of differentiation from preadipocytes [39]. Accordingly, the reduction in TG levels implies that ADL is able to suppress adipogenesis.

**Figure 4:**
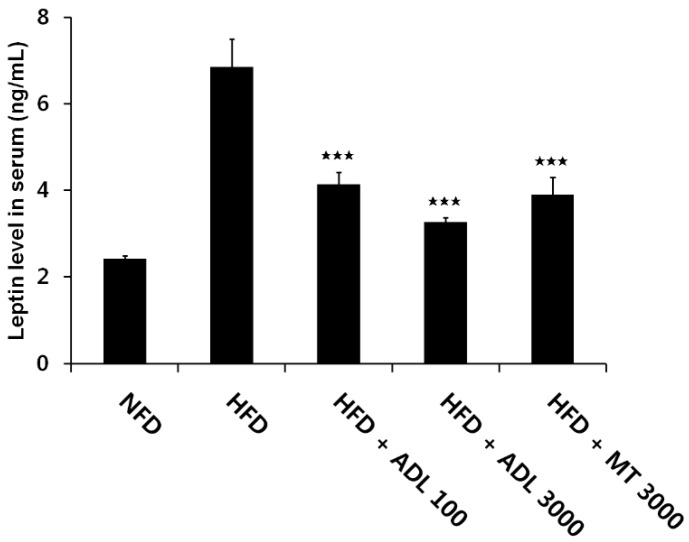
Effect of ADL on serum leptin. Mice were fed normal-fat diet (NFD), high-fat diet (HFD), HFD supplemented with 100 mg·kg^−1^·day^−1^ ADL (HFD + ADL 100), HFD supplemented with 3000 mg·kg^−1^·day^−1^ ADL (HFD + ADL 3000), and HFD supplemented with 3000 mg·kg^−1^·day^−1^ yerba mate (HFD + MT 3000) for 6 weeks. Serum samples were obtained from 15 h-fasted mice. Leptin was assayed by ELISA. Bars represent mean ± SD (*n *= 3). Statistical significance was evaluated by Duncan post-hoc test in ANOVA. ^
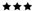
*p*^ < 0.001, compared to the high-fat diet group.

Adipocytes in adipose tissue secrete a variety of proteins, known as adipocytokines leptin, tumor necrosis factor-α, interleukin-6, resistin and adiponectin [40]. Among these, serum leptin is positively correlated with adiposity and body weight changes in both humans and rodents [41]. Serum leptin levels have been shown to be proportional to body fat mass [42,43]. In this study, serum leptin levels were significantly lower in both HFD + ADL (100 mg·kg^−1^·day^−1^) and HFD + ADL (3000 mg·kg^−1^·day^−1^) mice than in HFD mice (Figure 4). These results suggest that the reduction of serum leptin by ADL may be attributable to decreased fat accumulation in adipose tissue, decreased body weight gain and lower organ weight. Accordingly, we suggest that in mice administered ADL, the decrease in serum leptin is a causal factor in the suppression of HFD-induced obesity.

## 4. Conclusions

In this study, ADL administered to HFD-fed mice prevented the typical increases in body weight and organ weight, particularly epididymal adipose tissue weight, a marker of visceral fat [44,45,46]. The prevention of body weight is likely caused by inhibition of accumulation of epididymal adipose tissue. The decrease in organ weight results from a decrease in the size and number adipocytes through downregulation of genes related to adipose metabolism, such as PPARG, CEBPA and LPL [6,18]. Moreover, in mice fed HFD + ADL, serum TG and leptin were reduced, relative to the HFD group. TG synthesis is a marker of adipocyte differentiation and leptin is positively correlated with adiposity and changes in body weight. Taken together, these results suggest that ADL disrupts the development of HFD-induced obesity via downregulation of genes involved in adipose metabolism in epididymal adipose tissue. In recent studies, it was reported that visceral adiposity was considered as a key factor for obesity-associated diseases [47,48]. Surgical removal of visceral fat improved hepatic insulin action [49,50,51] and reduced excessive inflammatory cytokines [49,52]. Accordingly, we suppose that ADL may play a very important role in prevention and treatment of visceral obesity-associated diseases and symptoms, because ADL is involved in the reduction of the visceral fat. In conclusion, we suggest that ADL, a natural, insect-derived product, exhibits a strong potential for development as a nutritional supplement or pharmaceutical intervention against obesity.
